# Chimeric antigen receptor (CAR)-T-cell therapy for glioblastoma: what can we learn from the early clinical trials? A systematic review

**DOI:** 10.1093/noajnl/vdaf115

**Published:** 2025-06-03

**Authors:** Justine Tin Nok Chan, James Henley-Waters, Saeed Kayhanian

**Affiliations:** Fitzwilliam College, University of Cambridge, Cambridge, UK; School of Clinical Medicine, University of Cambridge, Cambridge, UK; Fitzwilliam College, University of Cambridge, Cambridge, UK; School of Clinical Medicine, University of Cambridge, Cambridge, UK; Department of Clinical Neurosciences, University of Cambridge, Cambridge, UK; Fitzwilliam College, University of Cambridge, Cambridge, UK

**Keywords:** glioblastoma, chimeric antigen receptor, CAR-T therapy, clinical trials, systematic review

## Abstract

**Background:**

Glioblastoma is a malignant brain tumor with poor outcomes. Chimeric antigen receptor-T (CAR-T)-cell therapy is a possible new intervention in solid tumors using T cells engineered with cancer-specific antigens. This approach is challenged by limited T-cell trafficking to solid tumors, the immunosuppressive tumor microenvironment, and glioblastoma-specific uncertainties. Several first-in-human trials have now trialed CAR-T therapy for glioblastoma. We undertake a systematic review of these Phase I trials to draw early lessons about the safety and feasibility of this approach.

**Methods:**

Systematic review of all published clinical trials using CAR-T therapy for glioblastoma on July 31, 2024, from 5 databases.

**Results:**

Thirteen published studies of Phase I trials of CAR-T therapy for glioblastoma (*n* = 128 patients). Six molecular targets were used, most commonly EGFR family (7 studies) and IL13a2 (4 studies). There were 141 severe adverse effects (SAEs) and 2 dose-limiting toxicities. SAEs were most commonly neurological or hematological, and most commonly observed with doses over 1 × 10^7^ cells. Routes of delivery included intravenous, intraventricular, intracavitary, and intratumoral. Several participants across trials demonstrated transient responses, but efficacy across trials was difficult to compare, given heterogeneous reporting of outcomes.

**Conclusions:**

Several CAR-T strategies have now been trialed preliminarily for glioblastoma. There appears to be a signal of efficacy, with 56 of 128 reported patients demonstrating at least some measure of response. Central delivery of CAR-T cells appears safe with doses up to 2.5 × 10^7^ cells well-tolerated. Subsequent CAR-T trials should standardize reporting of outcomes for better comparison across trials.

**Key Points**

- Systematic review of chimeric antigen receptor-T (CAR-T) therapy in all 13 published glioblastoma Phase I trials.
- CAR-T therapy in glioblastoma is well tolerated with few dose-limiting events.
- Reported outcomes should be standardized to determine efficacy across early trials.

**Importance of the Study**

Chimeric antigen receptor-T (CAR-T) therapy is a promising new therapeutic modality being investigated across several solid cancer types. Thirteen Phase I studies have investigated the safety and feasibility of CAR-T therapy for the treatment of glioblastoma, using several different molecular antigen targets. Here we summarize the design and results from these first-in-human studies and draw key conclusions about the safety and feasibility of this approach, to guide the design and interpretation of future studies. Importantly, we find that centrally delivered CAR-T therapies appear to be well tolerated across a range of antigen targets, with dose-limiting toxicities appearing at doses over 2.5 × 10^7^ cells. Heterogeneity in reporting outcomes across these studies has limited cross-study comparisons for any signals of efficacy and is highlighted for the design of future trials in this area.

Glioblastoma is the most common malignant brain tumor in adults, with an overall survival (OS) of <15 months.^1^ One promising potential new therapeutic modality is chimeric antigen receptor (CAR)-T-cell therapy. This is a form of immunotherapy that collects T cells and modifies them by transferring a transgene coding for a chimeric receptor specific for a predetermined tumor-associated antigen.^2^ When reintroduced to the patient, these cells target tumor cells for immune-mediated destruction. CARs are composed of an extracellular domain (the single-chain variable fragment from a monoclonal antibody that recognizes the target antigen) and an intracellular domain, which activates and drives T-cell function, including target cell killing, which has been iterated in successive generations of CAR-T-cell therapy.^2^

The success of CAR-T therapy in treating hematological malignancies has sparked interest in its use for solid tumors, particularly for difficult-to-treat brain tumors such as glioblastoma,^2^ given possible advantages in blood–brain barrier penetration through immune cell trafficking and the use of direct killing,^3^ thus overcoming cancer-induced immune suppression in glioblastoma.^4^

To continue to progress the rational development of the next generation of CAR-T-based strategies for glioblastoma, it is important to draw lessons from key aspects of the feasibility and safety from across the early trials of this therapy. We present a systematic review of the clinical trials completed to date of CAR-T for glioblastoma.

## Materials and Methods

### Criteria for Considering Studies for This Review

This systematic review was performed following the Preferred Reporting Items for Systematic Reviews and Meta-Analysis (PRISMA) guidelines, the checklist for which is available in Supplementary Material 1. The protocol was registered on PROSPERO (ID: 646572). Studies were selected based on the following inclusion criteria: (1) clinical trials, including Phases I–IV, single-arm or multiple-arm studies, (2) studies with adult human glioblastoma patients, (3) studies examining CAR-T-cell therapy, and (4) studies including at least one of the following reported outcomes: treatment response, survival, or toxicity/side effects. The following exclusion criteria were applied: (1) reviews, meta-analyses, editorials, cost reporting, conference abstracts, (2) non-English language.

### Search Methods for Identification of Studies

Two authors (J.C. and J.H.) performed a search of Embase/Ovid, PubMed/Medline, CENTRAL/Cochrane Register, Clinicaltrials.gov, and Web of Science on July 31, 2024. Search keywords of “glioblastoma,” “adoptive immunotherapy,” and “chimeric antigen receptors,” and their synonyms were used in “AND” and “OR” combinations. Nonhuman studies, systematic reviews, and meta-analyses were excluded. No time or language restriction was imposed in the search strategy. A full version of the search strategy is included in Supplementary Material 2.

### Data Collection and Analysis

#### Selection of studies.—

Searches were imported into Covidence (2024), and duplicates were removed. Two independent reviewers (J.C. and J.H.) applied inclusion and exclusion criteria to identify studies for data extraction. A third independent reviewer (S.K.) resolved any disagreements.

#### Data extraction and management.—

Two independent reviewers (J.C. and J.H.) extracted the following information from each study: authors, year, and journal of publication; patient numbers, inclusion, and exclusion criteria; therapy dose, route, and type; follow-up time and outcomes. Primary outcomes to be assessed were safety and toxicity, specifically relating to dose-limiting events, and all side/adverse effects reported. Secondary outcomes included survival (progression-free survival [PFS] and OS) and treatment response. After extraction, the data were compared by the 2 reviewers to identify any conflicts. Studies were compared by methodology to ascertain quality differences, but not formally assessed using a risk of bias tool, due to their exploratory nature.

### Analysis

Studies were summarized descriptively and by type of therapy. Where possible, studies were compared by OS and PFS. All statistical analyses were performed using R statistical package v3.4.1 http://www.r-project.org (accessed on February 10, 2025).^5^

## Results

### Description of studies

#### Results of the search

**Figure 1.**
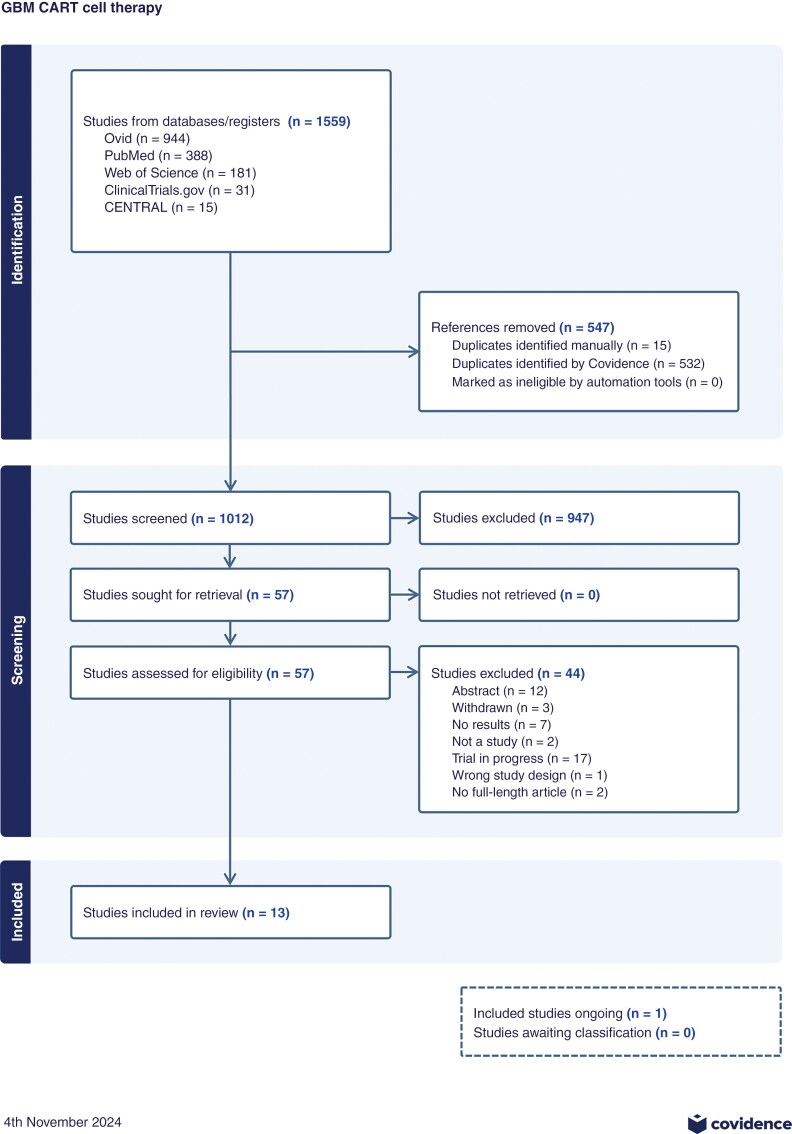
PRISMA flow diagram. Abstract identification, screening, and full-text inclusion were performed independently by 2 authors. From 1559 studies identified by our search strategy, 13 studies were included in this review after applying inclusion and exclusion criteria to include clinical trials of adult glioblastoma patients in CAR-T-cell therapy trials.

A total of 1012 papers were identified after the removal of duplicates, from which 57 papers were assessed during full-text screening. Forty-four studies were removed for the following reasons: abstract only (12 studies), withdrawn studies (3 studies), trials with no results available (7 studies), not a clinical trial (2 studies), trial in progress with no interim results (17 studies), wrong study design (1 study), and no full-length article available (2 studies). Thirteen studies remained and were included in this review, of which 1 reports interim results of an ongoing trial. Only results from adult patients being treated for glioblastoma aged 18 and above were included, with 1 study also involving 4 pediatric patients out of the 8 total patients (Liu 2023).^6^Figure 1 shows the flowchart based on the PRISMA statement.

#### Included studies

A summary of trials included is found in Table 1. A total of 128 patients across 13 trials are included in this analysis. Patients were aged between 24 and 76, with 88 (68.8%) being male. Ten trials were based in the USA (107 patients),^7–16^ with the remaining 21 patients across 3 trials being conducted in China.^6,17,18^

**Table 1.** Studies included in this review (*n* = 13)

Study	NCT number	Location	Study start and end date (YYYY-MM)	Study funding	Total number of adult participants with glioblastoma	Study population	Route of administration
Choi 2024	NCT05660369	United States	2023-03 to 2023-07	Academic	3	Recurrent/progressive	Intraventricular
Bagley 2024.1	NCT03726515	United States	2019-03 to 2021-02	Commercial—Novartis, Academic	7	Newly diagnosed	Intravenous
Liu 2023	NCT03170141	China	2020-05 to 2020-08	Academic	8^b^	Recurrent	Intravenous (4) and intracavitary (2)
Brown 2022	NCT01082926	United States	2011-03 to 2013-03	Academic	6	Recurrent/progressive	Intracavitary
Lin 2021	NCT03423992	China	2018-03 to 2023-01	Not described	3	Recurrent	Intravenous
Guo 2019	NCT02937844	China	2016-07 to 2019-07	Not described	14	Recurrent	Intravenous (14/14) and intracranial (1/14)
Goff 2019	NCT01454596	United States	2012-05 to 2018-11	Academic	18	Recurrent	Intravenous
O’Rourke 2017	NCT02209376	United States	2014-11 to 2018-04	Academic	10	Recurrent/newly diagnosed	Intravenous
Ahmed 2017	NCT01109095	United States	2011-07 to 2014-04	Academic	10	Recurrent	Intravenous
Bagley 2024.2	NCT05168423	United States	2023-06 to 2024-02^a^	Commercial—Kite Pharma, Academic	6	Recurrent	Intraventricular
Brown 2024	NCT02208362	United States	2015-05 to 2023-12	Commercial—Mustang Bio, Academic	41	Recurrent/progressive	Intratumoral, intraventricular and dual ICT/ICV
Brown 2015	NCT00730613	United States	2002-02 to 2011-08	Academic	3	Recurrent	Intracavitary and intratumoral
Landi 2023	NCT02664363	United States	2017-02 to 2019-09	Not described	3	Newly diagnosed	Intravenous

Abbreviation: NCT = National Clinical Trials.

^a^Interim end date of study in progress with interim results available.

^b^Four out of 8 total participants who were aged >18 in the study.

The recruited patients in each study ranged widely in age (28–76 years), but were similar in prior treatments received, with 66%–100% of patients having received at least 1 surgical resection, and all patients except from 1 trial (Brown 2024) having received temozolomide.^13^ Six of 13 trials’ patients had reported additional pharmaceutical treatment in addition to this.^6–8,10,11,17^ Patients in 10 trials all had received prior radiotherapy,^7–12,14,15,17,18^ with 1 trial having had 3/4 patients receiving prior radiotherapy.^6^

All studies provided information on adult recurrent glioblastoma patient survival via case-fatality rates. Ten studies reported OS,^6,8,10–14,16–18^ and of these 4 reported PFS.^6,8,11,16^ Of the 12 studies that included an imaging measure for clinical response to therapy, all used MRI (12/12),^6–14,16–18^ with 2 studies using both MRI and PET to evaluate tumor changes in response to CAR-T therapy. 10/13 studies reported toxicity/side effects for glioblastoma treatment in our patient population.^7–12,14,15,17,18^

### Synthesis of Results

#### By intervention.—

**Figure 2.**
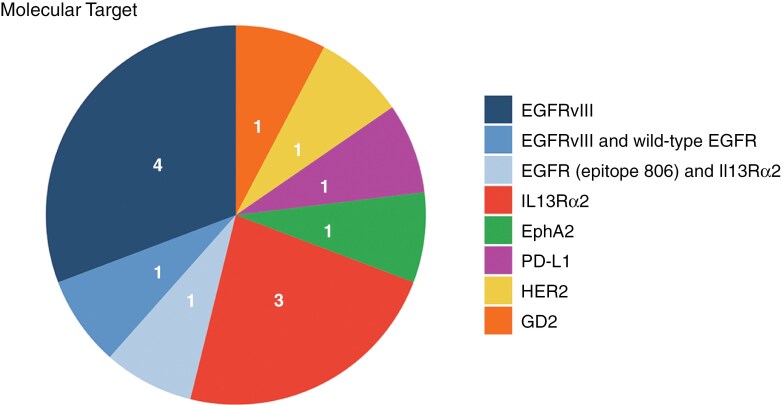
Pie chart showing targets of CAR-T-cell therapies used in studies included in this review (*n* = 13). EGFR = epidermal growth factor receptor.

Table 2 summarizes the interventions used in each study. Across 13 reports, 7 studies used targets within the EGFR family, 4 against EGFRvIII (epitope 806),^8,11,12,15^ 1 against HER2,^16^ 1 against EGFRvIII and the wildtype EGFR,^7^ and 1 against both EGFRvIII and IL13Rα2.^9^ Of the remaining studies, there were 3 more studies solely targeting IL13Rα2,^10,13,14^ and 1 study for each of GD2,^6^ EphA2,^17^ and PD-L1.^18^ This is depicted in Figure 2.

**Table 2.** Details of interventions of each study included in this review (*n* = 13)

Study	Target of CAR-T cells	Source of CAR-T cells	Other features of CAR-T cells	Dose level(s) with units	Time between diagnosis and T-cell infusion (median)	Time between diagnosis and T-cell infusion (range)	Pre-adjuvant therapies	Concurrent and adjuvant therapies
Choi 2024	EGFRvIII and wild-type EGFR	Autologous	Secretion of a T-cell–engaging antibody molecule	i.v.: 10 × 10^6^ cells	12 months	6–20 months	NA	Anakinra (100 mg every 6 h) for fever
Bagley 2024.1	EGFRvIII	Autologous	NA	i.v.: DL1: 2 × 10^8^ (*n* = 6) DL2: 4.65 × 10^7^ (*n* = 1)	NA	NA	Radiotherapy—40 Gy in 15 fractions over 3 weeks	Pembrolizumab (200 mg given 1 h before CAR-T) Pembrolizumab monotherapy as a fourth cycle after 3 cycles of CAR-T-EGFRvIII celll/combined with pembrolizumab therapy
Liu 2023	GD2	Autologous	CD28 transmembrane and cytoplasmic domains, co-stimulatory 4-1BB intracellular TRAF binding domain, CD3z chain intracellular domain, and an inducible suicide caspase 9 gene	i.v. and i.c.: DL1: i.v 1.3 × 10^8^, i.c 5 × 10^6^ (*n* = 1) DL2: i.v 1.4 × 10^8^ (*n* = 1) DL3: i.v 1.6 × 10^8^, i.c 6.4 × 10^6^ (*n* = 1) DL4: i.v 2.1 × 10^8^ (*n* = 1)	NA	NA	Lymphodepletion from 4 days before to 2 days before treatment	NA
Brown 2022	IL13Rα2	Allogeneic	IL13-zetakine + GRm13Z40-2 T cells	i.c.: 1 × 10^8^ cells	24 months (mean)	5.8–79 months (from diagnosis to enrollment)	Daily dexamethasone	IL-2 (aldesleukin; 9 infusions ranging from 2500 to 5000 IU) Maintained on daily dexamethasone dose of ≥4 mg
Lin 2021	EphA2	Autologous	NA	i.v.: 1 × 10^6^ cells/kg	16 months	NA	Fludarabine (25 mg/m^2^, from 3 days before to 1 day before treatment) and Cyclophosphamide (250 mg/m2, from 2 days before to 1 day before treatment)	NA
Guo 2019	PD-L1	Autologous	Chimeric switch receptor (CSR) of extracellular domain of PD1 and transmembrane and cytoplasmic domain of CD28	i.v and dual i.v/i.c i.v.: up to 1 × 10^9^ cells, with dose gradually increasing i.c.: DS (*n* = 1): 5.0 × 10^6^ (week 1), 1.5 × 10^6^ (week 2), 3.0 × 10^6^ (week 3), 4.0 × 10^6^ (week 5)	NA	NA	NA	Saline infusion after CSR-T cells
Goff 2019	EGFRvIII	Autologous	NA	i.v.: DL1:1 × 10^7^ (*n* = 2) DL2: 3 × 10^7^ (*n* = 2) DL3: 1 × 10^8^ (*n* = 2) DL4: 3 × 10^8^ (*n* = 1) DL5: 1 × 10^9^ (*n* = 3) DL6: 2.48 × 10^9^ (*n* = 1) DL7: 3 × 10^9^ (*n* = 2) DL8: 1 × 10^10^ (*n* = 3) DL9: 6 × 10^10^ (*n* = 1) DL10: 3 × 10^10^ (*n* = 1)	15.7 months	11.6–20.6 months (IQR)	2 days of cyclophosphamde (60 mg/kg) and 5 days of fludarabine (25 mg/m^2^), before CAR-T	Low-dose intravenous interleukin-2 (IL-2) administration (72 000 IU/kg), within 24 h of cell transfer, continued every 8 h to tolerance
O’Rourke 2017	EGFRvIII	Autologous	NA	i.v.: DL1: 4.99 × 10^8^ (*n* = 1) DL2: 1.75 × 10^8^ (*n* = 1) DL3: 5 × 10^8^ (*n* = 8)	358 days	179–682 days	NA	NA
Ahmed 2017	HER2	Autologous	NA	i.v.: DL1: 1 × 10^6^/m^2^ (*n* = 2) DL2: 3 × 10^6^/m^2^ (*n* = 3) DL3: 1 × 10^7^/m^2^ (*n* = 3) DL4: 3 × 10^7^/m^2^ (*n* = 2) DL5: 1 × 10^8^/m^2^ (*n* = 3) DL6: 1 × 10^6^ + 3 × 10^6^ + 1 × 10^7^ + 3 × 10^7^ (*n *= 1)	14.65 months	11.3–27.2 months	NA	NA
Bagley 2024.2	EGFR (epitope 806) and IL13Rα2	Autologous	NA	intraventricular: DL1: 1 × 10^7^ cells (*n* = 3) DL2: 2.5 × 10^7^ cells (*n* = 3)	NA	NA	NA	NA
Brown 2024	IL13Rα2	Autologous	Hinge-optimized, 41BB-costimulatory CAR	Intratumoral, intraventricular + dual intratumoral/intraventricular: DS1: 2 × 10^6^ cells (week 1), 10 × 10^6^ (week 2), 10 × 10^6^ (week 3) DS2: 10 × 10^6^ cells (week 1), 50 × 10^6^ (week 2), 50 × 10^6^ (week 3) DS3: 20 × 10^6^ cells (week 1), 100 × 10^6^ (week 2), 100 × 10^6^ (week 3)	NA	NA	NA	NA
Brown 2015	IL13Rα2	Autologous	IL13(E13Y)-zetakine CD8 + CTL	i.c.: first 3 doses of 1 × 10^7^, 5 × 10^7^, and 5 × 10^8^ cells, followed by 9 additional infusions of 1 × 10^8^ cells per dose, except 1 patient who skipped day 1 of cycle 3 skipped due to transient worsening of headache (*n* = 3), intratumoral: up to 1 × 10^8^ cells per infusion (*n* = 1)	NA	NA	NA	NA
Landi 2023	EGFRvIII	Autologous	NA	i.v.: 4.5 × 10^6^ cells/kg	NA	NA	Standard of care radiotherapy and temozolomide, standard dose-intensified temozolomide at least 48 h before T cell infusion	Temozolomide: 1 month after T-cell infusion—cycles of SOC 5-day temozolomide at 150–200 mg/m^2^/day for the first 5-day cycle, followed by 200 mg/m^2^/day for 5 days every 28 days

Abbreviations: CAR-T cells = chimeric antigen receptor-T cells; DL = dose level; DS = dose schedule; EGFR = epidermal growth factor receptor; i.c. = intracavitary; i.v. = intravenous.

#### Side effects.—

**Figure 3:**
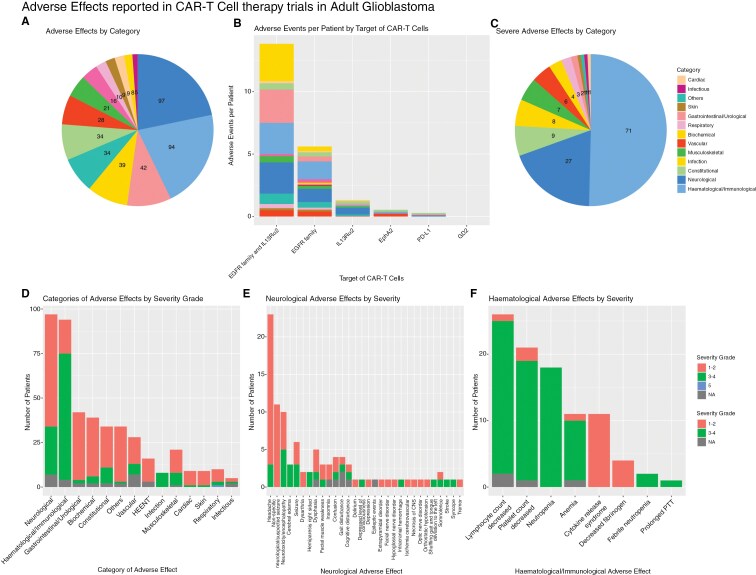
Side effects of CAR-T-cell therapies reported across 13 studies. (a) Side effects summarized by body system affected, showing the majority of side effects were neurological or hematological/immunological in nature. (b) Side effects summarized by target of CAR-T cells, divided by the number of patients receiving said targeted CAR-T-cell therapy, such that the number of side effects is adjusted for the different numbers of patients tested per CAR-T-cell target. This shows that the number of adverse events is highest for trials targeting both EGFR and IL3Ra2 antigens. (c) Side effects of severity grade 3–4 or above, deemed “severe,” summarized by body system affected. (d) Side effects summarized by body system affected, with color shading showing grade of severity (1–5). (e) Total numbers of each neurological side effect reported across all studies, with color shading showing grade of severity. (f) Total numbers of each hematological side effect reported across all studies, with color shading showing grade of severity.

The side effects reported by each study are summarized in Figure 3 (individual study data available in Supplementary Material 3 and aggregated in Supplementary Material 4). As shown in Figure 3a, most side effects are neurological (*n* = 97 events), followed closely by hematological effects (*n* = 94 events), then by gastrointestinal/urological (*n* = 42 events) and biochemical effects (*n* = 39 events). Of the neurological effects, the most common is headache (*n* = 23 events), then suspected seizures/nonspecific neurological events (*n* = 11 events), in addition to 7 confirmed epileptic events/seizures.

Figure 3b depicts the adverse events reported, grouped by the molecular target of the CAR-T-cell therapy. The distribution of adverse effects across body systems appears consistent regardless of the target, possibly with a slight propensity for neurological effects in trials targeting IL13Rα2 compared to those targeting the EGFR family. In contrast, there is a trend toward an increased proportion of adverse hematological effects in trials against the EGFR family of receptors compared to other trials.

Figure 3c depicts serious adverse events (grading of 3–4 or 5). A total of 141 severe adverse events were recorded in this grade, the majority of which were hematological/immunological (71), followed by neurological (27) and constitutional (9). Figure 3d summarizes all adverse effects regardless of severity. Although neurological side effects outnumbered hematological changes, there were proportionally more serious side effects of hematological nature than neurological ones.

There was 1 dose-limiting toxicity (grade 5, respiratory) reported by Goff 2019, at a dose level of 6 × 10^10^ T cells, which was the highest order of T cells tested across all trials.^11^ The patient developed acute dyspnea and hypoxia requiring mechanical ventilation and eventually succumbed to pulmonary edema.^11^ A second dose-limiting toxicity (grade 3, fatigue, generalized muscle weakness, and anorexia) was reported by Bagley 2024.2, at a dose level of 2.5 × 10^7^ T cells.^9^ The patient improved with dexamethasone.

As shown in Figure 3e, the most serious neurological side effect was neurotoxicity/encephalopathy, of which 50% (*n* = 5/10) were graded 3–4 in terms of severity. Other serious side effects included cerebral edema, seizures, and forms of muscle weakness including hemiparesis and facial muscle weakness. There were several subcortical (cerebellar/basal ganglia) events, including gait disturbance, extrapyramidal disorders, and shuffling gait with tongue deviation.

Figure 3f shows the array of hematological side effects, which shows that the majority of side effects are at least grade 3–4. Most of these are related to the depletion of immune cells, namely lymphocytes (mostly neutropenia) and platelets. Some patients also experienced anemia. Only 11 events of cytokine release syndrome were recorded, all of which were severity grade 1–2.

#### Survival and treatment response.—

Limited data were available on survival outcomes and treatment response. The majority of studies reported OS from infusion/registration with the trial, as opposed to from initial glioblastoma diagnosis, except Brown et al., which only reported OS from diagnosis.^14^

**Figure 4.**
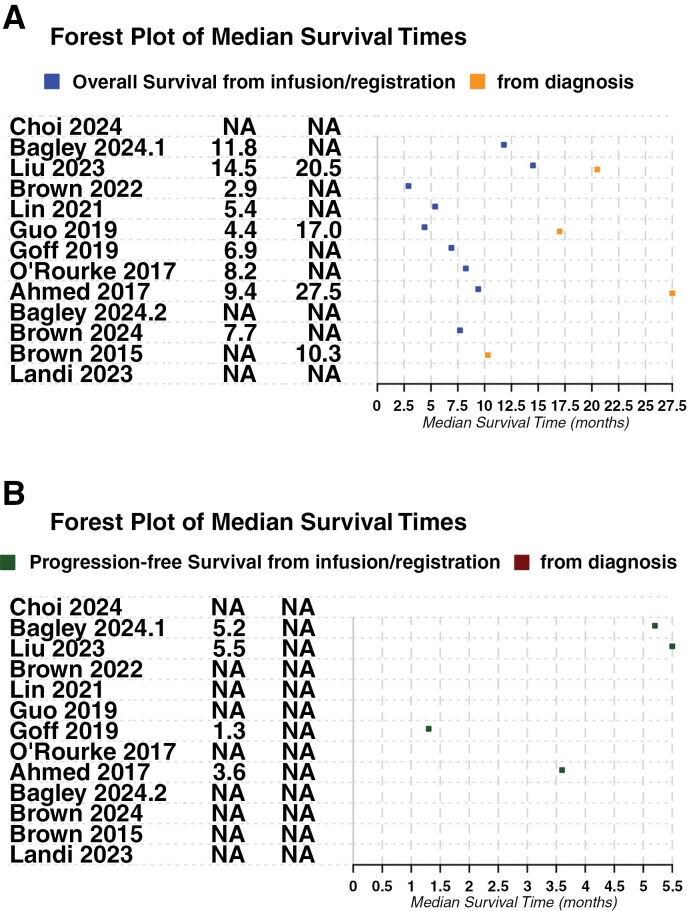
Survival odds reported by studies. NA = not applicable (no survival odds reported by study). (a) Overall survival represented as median survival time in months, either reported from infusion of CAR-T cells/registration of patients in the trial, and/or from initial diagnosis of glioblastoma. (b) Progression-free survival, represented as median survival time in months, either reported from infusion of CAR-T cells/registration of patient in the trial, and/or from initial diagnosis of glioblastoma.

As depicted in Figure 4a, most trials had OS between 2.9 and 9.4 months. Two studies had longer OS from infusion/registration—Bagley et al. (11.8 months)^8^ and Liu et al. (14.5 months).^6^ These studies also had longer PFS times than the 2 other studies that also reported PFS (Figure 4b).^11,16^ However, this may be due to patient selection criteria rather than the true effect of immunotherapy, as discussed later.

Brown et al. reported the shortest OS of 2.9 months (Figure 4a).^10^ This was also the only study that used allogeneic T cells. Of the remaining 3 studies of IL13Rα2 in total, Bagley et al. did not report OS/PFS as the trial has not completed yet,^9^ and Brown et al. reported OS 10.3 months from relapse.^14^ Brown et al. reported an OS of 7.7 months from the date of surgery.^13^

All studies except Landi used MRI to provide a response by imaging.^15^ To judge responses on imaging, mRANO (2 studies)^9,13^ or iRANO (3 studies) was used.^12,17,18^ Overall, there was no sustained response to therapy demonstrated by imaging. To measure tumor/immune response, 5 studies used tumor biopsy with genetic or biochemical staining for the target of interest,^7,8,10,12,14^ 1 study used CSF analysis for the target of interest,^7^ and 5 studies used CSF/periphery levels of cytokines and immune effectors as an indication of CAR-T-cell efficacy.^6,9,12,17,18^ To measure engraftment/uptake/infiltration of T cells, 4 studies used brain biopsy,^8,10,12,14^ liquid biopsy from CSF,^7,9^ and/or blood.^6–9,11,12,16,17^

## Discussion

### Summary of Main Results

We present a systematic review of all completed studies of CAR-T for glioblastoma in adult populations. Across 13 Phase I studies, there were 141 serious adverse events related to the intervention and 2 dose-limiting toxicities that required the intervention to be stopped. Most adverse effects were immunological or neurological in nature. Signals of efficacy are difficult to compare across trials, given the varying secondary outcome measures collected but, overall, there appears to be a suggestion of transient tumor response that did not persist significantly.

#### Safety.—

Specific adverse events may arise due to “on-target, off-tumour toxicity,” when targets are present in healthy tissue, or “off-target” toxicity, when the immunotherapy is cross-reactive against nontarget antigens expressed natively. Most adverse events were hematological, consistent with the mechanism of intervention, and often related to deranged laboratory blood results. Neurological adverse events were the second most common, with headache being the most common neurological side effect. This is consistent with the target and methods of CAR-T-cell therapy, with no sign of obvious on-antigen, off-target effects.

The 2 dose-limiting toxicities were at relatively higher orders of doses (1 × 10^10^ and 1 × 10^7^ cells).^9,11^ Although each study did not individually report any relationship between dose and adverse effect, there may have been a slight dose–adverse effect relationship, as seen in Supplementary Material 4, as 6 of the 7 studies that reported adverse effects of grade 3 and above used dose orders of 1 × 10^7^ or above,^8–12,14^ whereas the remaining 2 studies that only reported adverse effects of grades 1–2 used lower intracranial dose orders of 1 × 10^6^^17,18^ although one of these used IV doses of up to 1 × 10^9^.^18^ However, not enough data are available to make more conclusive statements about any relationship between dose and side effects.

Some studies used locoregional delivery to reduce the delivery of CAR-T cells to off-target organs. Although there was no clear safety benefit of such locoregional delivery over peripheral delivery, this lack of expected benefit may have been due to some degree of leakage to the periphery, as CAR-T cells were detected in the blood by both Brown^13^ and Brown et al..^10^ Other studies using central delivery did not report this.^9,14^ There may also have been some correlation with dose, as reported by Bagley, Brown, and Goff,^8,11,14^ in which the most severe toxicities occurred at the higher end of the dose schedules (10^8^ T cells or more). Nonetheless, most studies concluded that their therapy was relatively safe.

There may have been some relationship between the type of toxicity and the target. As stated earlier, we showed that there may be a higher proportion of neurological effects in anti-IL13Rα2 therapy and a higher proportion of hematological effects in anti-EGFR family trials. This is consistent with the EGFR receptor family being expressed widely in the body including in vascular endothelial cells. Moreover, the majority of IL13Rα2 trials used intracavitary/intratumoral routes of delivery, explaining their more nervous-system-restricted effects, in contrast to the EGFR CAR-T-cell therapies, which were mainly delivered intravenously.

#### Route of delivery.—

There is some limited evidence that direct methods of treatment delivery (intracavitary, intraventricular, or intratumoral) may be more successful at allowing CAR-T cells to affect their function than intravenous delivery. Brown et al. showed that CD8 + T cells were higher at the tumor injection site than at a more distal tumor biopsy site after intracavitary injection,^10^ Choi et al. showed that the percentage of CAR-T cells in the blood was consistently lower (0%–2%) than in CSF (~70%) after intraventricular injection,^7^ and Bagley et al. showed that initial expansion of CAR-T cells was higher in CSF than in peripheral blood of all patients.^9^

The findings from intravenous-only studies are consistent with the suggestion for the superiority of direct delivery methods. One intravenous-only study detected little to no CAR-T cells in the brain in 6 of 7 patients alongside low peripheral engraftment.^8^ However, this conclusion is limited by insufficient central sampling. Other studies investigating intravenous-only routes only used methods to detect CAR-T cell DNA in the blood,^16,17^ and therefore were only able to demonstrate effective peripheral engraftment.

To more explicitly compare routes, a few studies combined both, although the comparison is limited by the heterogeneous manner in which this was done across studies.^6,13,18^ In a larger trial by Brown et al. of 41 adult recurrent glioblastomas, up to 19/41 adult patients achieved stable disease.^13^ Those on regimens using dual intratumoral/intraventricular delivery of pretreated T cells had statistically better survival than other arms of single intratumoral or intraventricular delivery, even after adjusting for baseline and tumor differences. However, this response did not seem to be mediated by higher CAR-T levels in the CSF, as this did not differ between treatment arms. In Guo et al.^18^, 1/14 patients had additional intracranial infusions after intravenous infusions. The authors suggested that the greater IFNγ and IL-6 levels in the plasma in this patient could imply “higher bioavailability following intracranial injection than that following intravenous administration.” However, this did not correlate with any clinical response and the authors concluded that their study was underpowered to detect any differences.

#### Lymphodepletion.—

However, factors other than the infusion route, such as persistence, may also influence the success of therapy. Two studies suggested that there was poor engraftment and expansion of CAR-T cells after infusion. This was potentially attributed to the lack of lymphodepletion in their protocols.^8,14^ Indeed, studies that did employ lymphodepletion (using various mechanisms including cytotoxic therapies like fludarabine and cyclophosphamide) showed some degree of expansion, if transient.^6,11,17^ However, O’Rourke noted that although no lymphodepletion was used, some patients were lymphopenic to begin with, but no correlation between peak engraftment and lymphocyte count was noted.^12^ Guo et al. was the only protocol to require a minimum lymphocyte count, but did not report any problems with engraftment.^18^

#### Persistence

##### Immune exhaustion.—

Across all studies, trials suggest that there is poor persistence of CAR-T cells even if they successfully engraft and expand. Most studies show that there is a rise in detectable CAR-T cells early after infusion, which peaks within 7–14 days, eventually becoming undetectable or at very low levels by 1 month.^6–12,17^ Many studies attributed this to the immunosuppressive tumor microenvironment of glioblastoma.

One key mechanism is by expressing immune checkpoint ligands that downregulate attacking T cells, for example, by activating the PD-1/PD-L1 cascade.^19^ This would explain the rapid but transient tumor reduction and/or necrosis at the site of T-cell activity in many trials based on radiographic imaging,^7,10^ or the initial decrease in target antigen.^6–8^ Bagley et al. showed that there was an increase in exhaustion markers after treatment,^8^ while O’Rourke et al. showed that there was upregulation of adaptive resistance mechanisms, “including up-regulation of IDO1 and PD-L1.”^12^ Promisingly, Brown et al. showed that an arm of treatment using T cells enriched with naïve T cells (as opposed to central memory T cells) had a more balanced proportion of CD4+and CD8+ subsets, memory markers, and reduced expression of senescence marker CD57.^13^ Using these cells, patients had increased IFNγ-pathway-related cytokines/chemokines, which correlated with better survival in those with immunologically “cold” tumors.

Interestingly, in Brown et al., CAR-T levels in the blood but not in the CSF showed a significant positive correlation with LAG3 and a significant negative correlation with exhaustion markers PD-1 and CD57.^13^ This seemingly paradoxical relationship is relevant clinically as glioblastoma is known to induce T-cell expression of LAG3 and PD-1 and downregulate pro-inflammatory pathways such as IFN-γ. The authors did not examine any relationship between this and the treatment arm, which could be worth considering whether this has any effect on avoiding immunosuppression.

##### Noncancer TME cells.—

However, Bagley et al. did not report any improvement with the concurrent administration of Pembrolizumab, an anti-PD1 monoclonal antibody that aims to reduce tumor immune evasion.^8^ This could be because additional immunosuppressive mechanisms like tumor-associated macrophages and regulatory T cells were upregulated by CAR-T-cell therapy.

For example, regulatory T cells, myeloid-derived suppressor cells, and tumor-associated macrophages have been found to constitute up to 30% of glioblastoma tumor mass.^19^ Indeed, O’Rourke et al. also showed that there was “recruitment of IL-10–secreting, FoxP3-expressing regulatory T cells.”^12^ Overall, this shows that current CAR-T therapy is limited by the multiple resistance mechanisms that may be raised in response to 1 arm of treatment.

Other factors contributing to immune escape include the levels of CD3 + T cells in the tumor before any treatment. Brown et al. demonstrated that independent of the delivery method, levels of T cells (CD3+) in the tumor before any treatment are correlated with a significant survival benefit.^13^ This alludes to more local factors that contribute to the success of CAR-T-cell therapy.

##### Target heterogeneity.—

Most studies were able to demonstrate that the target antigen decreased in expression after CAR-T-cell therapy, including those targeting EGFRvIII,^8,12^ GD2,^6^ and IL3Ra2.^14^ Two studies showed that there was no change in target antigen expression of IL3Ra2,^10^ and EGFR.^12^ However, despite these decreases, there was no correlation with clinical benefit. One reason for such resistance is that glioblastoma is known to have target heterogeneity, in which there exists variability in mutations and genetic alterations within different regions of the same tumor. For example, subsets of cells may harbor mutations in genes like EGFR, PTEN, or TP53, while others do not.^20^ In keeping with this, Brown et al. showed that in recurrent tissue adjacent to the T-cell injection site, IL13Rα2 expression was decreased compared to pre-therapy levels, suggesting that the recurrence/progression of patients was due to IL13Rα2-negative or low tumor cells.^14^

### Quality of the Evidence

This review included 13 Phase I/II trials of 128 participants. In general, study results were relatively consistent in the transient efficacy of CAR-T-cell therapy with poor long-term benefit, with a few exceptions that were still stable after the end of the trial.

Across all trials, there was poor methodological consistency, making it difficult to draw conclusive comparisons about this. In particular, there were differences in patient selection criteria. It was reported that Bagley et al.^8^ and Liu et al.^6^ had better outcomes (OS from infusion/registration) than other studies reporting similar metrics. The former used CAR-T-cell therapy targeting EGFRvIII in combination with Pembrolizumab, while the latter used fourth-generation CAR-T cells against GD2. Although there may be some merit accounted to the former’s use of combination immunotherapy with chemotherapy or the latter’s relatively novel target antigen, their relative success may be due to their patient selection criteria being less stringent on the severity of the disease. For example, Bagley et al. accepted “newly diagnosed, histologically confirmed GBM,”^8^ and Liu et al. enrolled any patient with “recurrent glioblastoma or brain tumor patients with measurable tumors.”^6^ In contrast, most other studies explicitly required recurrent/refractory glioblastoma, with some also requiring specific late-stage disease seen in radiology/histology.^7,9–14,16–18^

There were also differences in clinical endpoints, with a variety of survival endpoints including OS from diagnosis, from infusion, from registration, and PFS. Different studies used different ways of measuring CAR-T-cell engraftment, persistence, bioactivity, and efficacy. Follow-up durations, where reported, were inconsistent across studies, ranging from 29 days to 59 months. This made it difficult to compare trials directly and highlights the need for standardization of reporting even of early clinical trials in this field.

Of note is that most studies used peripheral levels of IFNy and other type I cytokines like IL-6 and TNFa as a signature of the bioactivity of CAR-T cells. This was consistent with the tumor responses seen on imaging, in that there was a rise and peak followed by a fall.^6,8,12,17,18^ However, there are several limitations to using this as a proxy for CAR-T-cell efficacy. First, this only informs on T-cell activity and is not specific for CAR-T cells. Second, this is only a readout of activity in the periphery and does not necessarily correlate with their activity in the local tumor microenvironment. Finally, in glioblastoma, interferon signaling may be abrogated, such that the tumor often exhibits defective responses to IFN-γ signaling, even when the ligand binds to its receptor, which may facilitate immune evasion or even promote tumor growth. This is consistent with how Bagley et al. reported that the intensity of the IFN-related signature in T cells was positively correlated to the time from tumor progression to death.^8^

### Future Trials

Ongoing trials continue to examine some of the targets presented in this review, including EGFRvIII and IL3Ra2. We find good safety in trials examining direct delivery of the therapy to the central nervous system. Future trials should address the possible reasons for therapy nonsuccess raised in this review, including modifying T cells to address the cold immune environment, target heterogeneity, and immune exhaustion.

### Potential Biases in the Review Process

We present a systematic review of CAR-T-cell therapies. Although independent screening and data extraction by two reviewers were carried out, we note that we only searched English-language bibliographic and clinical trial databases and did not look at other forms of databases of unpublished literature or non-English studies. This may be significant as at least 2 studies reported here were based in China. Furthermore, we could not obtain comprehensive data from trials that were only registered on Clinicaltrials.gov and not published elsewhere and could only obtain information on study registration and basic patient outcomes. Liu et al. recruited a cohort of mixed adult and pediatric patients and hence toxicity information could not be obtained specifically for the adult cohort of interest.^6^

### Comparison to Literature

Our study agrees with the literature that CAR-T-cell therapy is a promising avenue of immunotherapy in glioblastoma but that current methods are limited in addressing immune evasion. Previous reviews have been performed, but we provide a timely update including 5–7 more articles showing new results.^21,22^ One meta-analysis has been attempted in the past, but as discussed earlier, most of the studies differed greatly across many methodological characteristics, rendering any meta-analysis unreliable. Nonsystematic reviews have been performed, highlighting in particular the success of preclinical trials.^23–25^ Our review shows the challenges of translating this into clinical success and highlights the key limitations that need to be overcome by CAR-T-cell design.

## Conclusions

CAR-T therapy is a promising new therapeutic modality being investigated across several solid cancer types. Thirteen Phase I studies have investigated the safety and feasibility of CAR-T therapy for the treatment of glioblastoma, using several different molecular antigen targets. Across trials, CAR-T therapy appears to be well tolerated even when delivered centrally but there is a suggestion of dose-limiting toxicity above 2.5 × 10^7^ cells. Trial participants demonstrated transient responses to CAR-T treatment and there were isolated reports of sustained response. The interpretation of efficacy across trials is limited by intertrial differences in initial cohort selection and reporting of outcomes. Future clinical trials should build on this available evidence on dose and route safety of CAR-T therapy for glioblastoma, while using standardized reporting measures, such as 12-month OS, to aid comparison across early trials to evaluate the variety of molecular targets being investigated.

## Supplementary Material

vdaf115_suppl_Supplementary_Materials

## Data Availability

Data of the third-party data analyzed will be made available upon reasonable written request to the authors.
