# Supplementary material for: Chimeric antigen receptor (CAR)-T-cell therapy for glioblastoma: what can we learn from the early clinical trials? A systematic review
Source: Neurooncol Adv. 2025 Jun 3;7(1):vdaf115. doi: 10.1093/noajnl/vdaf115 (PMC12232909; doi:10.1093/noajnl/vdaf115)
Supplement: vdaf115_suppl_Supplementary_Materials [file vdaf115_suppl_supplementary_materials.docx]

**Supplementary data**

[Supplemental 1: PRISMA checklist for systematic reviews (full paper and checklist) 2](#_Toc190088783)

[Supplemental 2: Search strategy in full across Embase/Ovid, PubMed/Medline, CENTRAL/Cochrane Register, Clinicaltrials.gov and Web of Science on 31 July 2024 8](#_Toc190088784)

[Supplemental 3: Side effects reported by each study individually 10](#_Toc190088785)

[Supplemental 4: Side effects, summarised by severity of side effect from 1 to 5, per study. 16](#_Toc190088786)

#

# Supplemental 1: PRISMA checklist for systematic reviews (full paper and checklist)

PRISMA 2020 Checklist

| **Section and Topic** | **Item #** | **Checklist item** | **Location where item**  **is reported**  page 6 |
| --- | --- | --- | --- |
| **TITLE** | | |  |
| Title | 1 | Identify the report as a systematic review. | 1 |
| **ABSTRACT** | | |  |
| Abstract | 2 | See the PRISMA 2020 for Abstracts checklist. |  |
| **INTRODUCTION** | | |  |
| Rationale | 3 | Describe the rationale for the review in the context of existing knowledge. | 4 |
| Objectives | 4 | Provide an explicit statement of the objective(s) or question(s) the review addresses. | 4 |
| **METHODS** | | |  |
| Eligibility criteria | 5 | Specify the inclusion and exclusion criteria for the review and how studies were grouped for the syntheses. | 5 |
| Information sources | 6 | Specify all databases, registers, websites, organisations, reference lists and other sources searched or consulted to identify studies. Specify the date when each source was last searched or consulted. | 5 |
| Search strategy | 7 | Present the full search strategies for all databases, registers and websites, including any filters and limits used. | 5 |
| Selection process | 8 | Specify the methods used to decide whether a study met the inclusion criteria of the review, including how many reviewers screened each record and each report retrieved, whether they worked independently, and if applicable, details of automation tools used in the process. | 5-6 |
| Data collection process | 9 | Specify the methods used to collect data from reports, including how many reviewers collected data from each report, whether they worked independently, any processes for obtaining or confirming data from study investigators, and if applicable, details of automation tools used in the process. | 6 |
| Data items | 10a | List and define all outcomes for which data were sought. Specify whether all results that were compatible with each outcome domain in each study were sought (e.g. for all measures, time points, analyses), and if not, the methods used to decide which results to collect. | 6 |
|  | 10b | List and define all other variables for which data were sought (e.g. participant and intervention characteristics, funding sources). Describe any assumptions made about any missing or unclear information. | 6 |
| Study risk of bias assessment | 11 | Specify the methods used to assess risk of bias in the included studies, including details of the tool(s) used, how many reviewers assessed each study and whether they worked independently, and if applicable, details of automation tools used in the process. | 6 |
| Effect measures | 12 | Specify for each outcome the effect measure(s) (e.g. risk ratio, mean difference) used in the synthesis or presentation of results. | 6 |
| Synthesis methods | 13a | Describe the processes used to decide which studies were eligible for each synthesis (e.g. tabulating the study intervention characteristics and comparing against the planned groups for each synthesis (item #5)). | 6 |
|  | 13b | Describe any methods required to prepare the data for presentation or synthesis, such as handling of missing summary statistics, or data conversions. | NA |
|  | 13c | Describe any methods used to tabulate or visually display results of individual studies and syntheses. | 6 |
|  | 13d | Describe any methods used to synthesize results and provide a rationale for the choice(s). If meta-analysis was performed, describe the model(s), method(s) to identify the presence and extent of statistical heterogeneity, and software package(s) used. | NA |
|  | 13e | Describe any methods used to explore possible causes of heterogeneity among study results (e.g. subgroup analysis, meta-regression). | NA |
|  | 13f | Describe any sensitivity analyses conducted to assess robustness of the synthesized results. | 6 |
| Reporting bias assessment | 14 | Describe any methods used to assess risk of bias due to missing results in a synthesis (arising from reporting biases). | 6 |
| Certainty assessment | 15 | Describe any methods used to assess certainty (or confidence) in the body of evidence for an outcome. | 6 |

| **Section and Topic** | **Item #** | **Checklist item** | **Location where item**  **is reported**  page 22  page 22  page 22 |
| --- | --- | --- | --- |
| **RESULTS** | | |  |
| Study selection | 16a | Describe the results of the search and selection process, from the number of records identified in the search to the number of studies included in the review, ideally using a flow diagram. | 7 |
|  | 16b | Cite studies that might appear to meet the inclusion criteria, but which were excluded, and explain why they were excluded. | NA |
| Study characteristics | 17 | Cite each included study and present its characteristics. | 7 |
| Risk of bias in studies | 18 | Present assessments of risk of bias for each included study. | 17-18 |
| Results of individual studies | 19 | For all outcomes, present, for each study: (a) summary statistics for each group (where appropriate) and (b) an effect estimate and its precision (e.g. confidence/credible interval), ideally using structured tables or plots. | Tables 1-2 |
| Results of syntheses | 20a | For each synthesis, briefly summarise the characteristics and risk of bias among contributing studies. | 8-11 |
|  | 20b | Present results of all statistical syntheses conducted. If meta-analysis was done, present for each the summary estimate and its precision (e.g. confidence/credible interval) and measures of statistical heterogeneity. If comparing groups, describe the direction of the effect. | 8-11 |
|  | 20c | Present results of all investigations of possible causes of heterogeneity among study results. | 17-18 |
|  | 20d | Present results of all sensitivity analyses conducted to assess the robustness of the synthesized results. | NA |
| Reporting biases | 21 | Present assessments of risk of bias due to missing results (arising from reporting biases) for each synthesis assessed. | 17-18 |
| Certainty of evidence | 22 | Present assessments of certainty (or confidence) in the body of evidence for each outcome assessed. | 17-18 |
| **DISCUSSION** | | |  |
| Discussion | 23a | Provide a general interpretation of the results in the context of other evidence. | 11-17, 20 |
|  | 23b | Discuss any limitations of the evidence included in the review. | 17-19 |
|  | 23c | Discuss any limitations of the review processes used. | 19-20 |
|  | 23d | Discuss implications of the results for practice, policy, and future research. | 19-21 |
| **OTHER INFORMATION** | | |  |
| Registration and protocol | 24a | Provide registration information for the review, including register name and registration number, or state that the review was not registered. | 6 |
|  | 24b | Indicate where the review protocol can be accessed, or state that a protocol was not prepared. | 6 |
|  | 24c | Describe and explain any amendments to information provided at registration or in the protocol. | NA |
| Support | 25 | Describe sources of financial or non-financial support for the review, and the role of the funders or sponsors in the review. | 22 |
| Competing interests | 26 | Declare any competing interests of review authors. | 22 |
| Availability of data, code and other materials | 27 | Report which of the following are publicly available and where they can be found: template data collection forms; data extracted from included studies; data used for all analyses; analytic code; any other materials used in the review. | 22 |

PRISMA 2020 for Abstracts Checklist

| **Section and Topic** | **Item**  **#** | **Checklist item** | **Reported**  **(Yes/No)**  Yes  Yes  No  No |
| --- | --- | --- | --- |
| **TITLE** | | |  |
| Title | 1 | Identify the report as a systematic review. | Yes |
| **BACKGROUND** | | |  |
| Objectives | 2 | Provide an explicit statement of the main objective(s) or question(s) the review addresses. | Yes |
| **METHODS** | | |  |
| Eligibility criteria | 3 | Specify the inclusion and exclusion criteria for the review. | Yes |
| Information sources | 4 | Specify the information sources (e.g. databases, registers) used to identify studies and the date when each was last searched. | Yes |
| Risk of bias | 5 | Specify the methods used to assess risk of bias in the included studies. | Yes |
| Synthesis of results | 6 | Specify the methods used to present and synthesise results. | Yes |
| **RESULTS** | | |  |
| Included studies | 7 | Give the total number of included studies and participants and summarise relevant characteristics of studies. | Yes |
| Synthesis of results | 8 | Present results for main outcomes, preferably indicating the number of included studies and participants for each. If meta-analysis was done, report the summary estimate and confidence/credible interval. If comparing groups, indicate the direction of the effect (i.e. which group is favoured). | Yes |
| **DISCUSSION** | | |  |
| Limitations of evidence | 9 | Provide a brief summary of the limitations of the evidence included in the review (e.g. study risk of bias, inconsistency and imprecision). | Yes |
| Interpretation | 10 | Provide a general interpretation of the results and important implications. | Yes |
| **OTHER** | | |  |
| Funding | 11 | Specify the primary source of funding for the review. | NA |
| Registration | 12 | Provide the register name and registration number. | NA |

# Supplemental 2: Search strategy in full across Embase/Ovid, PubMed/Medline, CENTRAL/Cochrane Register, Clinicaltrials.gov and Web of Science on 31 July 2024

Search date: 31 July 2024

MEDLINE

1. ("glioblastoma"[MeSH Terms]) OR (glioblastoma[Title/Abstract]) OR (glioblastoma*[Title/Abstract])
2. (CAR T-cell) OR (CAR-T cells) OR (CAR-T cell)
3. (chimeric antigen receptor) OR (chimeric antigen receptors) OR (Antigen Receptors, Chimeric[MeSH Terms]) OR ("receptors, chimeric antigen"[MeSH Terms])
4. ("immunotherapy, adoptive"[MeSH Terms]) OR (adoptive immunotherapy)
5. (Review[Publication Type]) OR (Meta-analysis[Publication Type])
6. 2 or 3 or 4
7. (1 and 6) not 5
8. (“Animals’[MeSH Terms]) NOT (“Animals”[MeSH Terms] AND “Humans”[MeSH Terms])
9. 7 not 8

Number of MEDLINE citations: 388

Ovid (Embase)

1. exp Glioblastoma/
2. adoptive immunotherapy/
3. (immunotherap* adj3 adoptiv*).tw.
4. Receptors, Chimeric Antigen/
5. ((chimeric* adj3 antigen receptor*) or (chimeric* adj3 immunoreceptor*) or (chimeric* adj3 T cell receptor*) or (chimeric* adj3 Tcell receptor*)).tw,kf,nm.
6. ((artificial* adj3 T cell receptor*) or (artificial* adj3 Tcell receptor*)).tw,kf,nm.
7. (exp animal/ or nonhuman/) not exp human/
8. meta analy:.mp. or review.pt. or systematic review.pt.
9. 2 or 3 or 4 or 5 or 6
10. 1 and 9
11. 10 not 7
12. 11 not 8
13. remove duplicates from 12

Number of Embase citations: 787 \

Clinicaltrials.gov

- Condition: glioblastoma; Glioblastoma multiforme; Glioblastomas
- Intervention: chimeric antigen receptor OR CAR-T cell OR CAR-T cells OR CAR-T cell immunotherapy OR Car-T Cell Therapy

Number of clinicaltrials.gov citations: 31

CENTRAL (Cochrane register)

1. exp “Glioblastoma”[MeSH Terms]
2. glioblastom* or GBM*
3. 1 or 2
4. exp “Immunotherapy, Adoptive”[MeSH Terms]
5. (immunotherap* near/3 adoptiv*)
6. (t-cell* near1 therap”) or (tcell* near/1 therap*)
7. exp “Receptors, Antigen, T-cell”[MeSH Terms]
8. (chimeric* near3 antigen receptor*) or (chimeric* near/3 immunoreceptor*) or (chimeric* near/3 T cell reporter) or (chimeric* near/3 Tcell receptor*)
9. (artificial* near/3 T cell receptor) or (artificial* near/3 Tcell receptor*)
10. 4 or 5 or 6 or 7 or 8 or 9
11. 3 and 10

Number of CENTRAL citations: 17

Web of Science

- (((((ALL=(glioblastoma)) AND ALL=(CAR-T OR "chimeric antigen receptor")) AND ALL=(trial OR therapy)) AND ALL=(clinical OR dose)) NOT ALL=(animal)) NOT DT=(Review)

Number of Web of Science results: 181

#

# Supplemental 3: Side effects reported by each study individually. Number reported in each study (severity grade, if available). NA = no severity grading data available.

| Adverse effect | Choi 2024 | Bagley 2024.1 | Liu 2023 | Brown 2022 | Lin 2021 | Guo 2019 | Goff 2019 | O’Rourke 2017 | Ahmed 2017 | Bagley 2024.2 | Brown 2024 | Brown 2015 | Landi 2023 |
| --- | --- | --- | --- | --- | --- | --- | --- | --- | --- | --- | --- | --- | --- |
| Neurotoxicity/encephalopathy | 1(3), 2(2) | 1(3-4) |  |  |  |  |  |  |  | 3(2), 3(3) |  |  |  |
| Headache | 1(1), 2(2) | 6(1-2) |  | 4(1), 1(2) |  |  |  | 4(2), 1(3) |  | 2(2) |  | 2(≥3) |  |
| Cytokine release syndrome | 2(1), 1(2) |  |  |  | 2(1) |  |  |  |  | 4(1), 2(2) |  |  |  |
| Chills/Rigors | 1(2) |  |  |  |  |  |  |  |  |  |  |  |  |
| Rash | 1(1), 1(1) | 5(1-2) |  |  |  |  |  |  |  |  |  |  |  |
| Dehydration | 1(2) | 1(1-2) |  | 1(3) |  |  |  |  |  |  |  |  |  |
| Dry mouth | 1(2) |  |  |  |  |  |  |  |  |  |  |  |  |
| Fatigue | 1(1), 1(3) | 7(1-2) |  | 1(2), 3(3) |  | 1(1) |  | 2(1) |  | 1(1), 1(3) |  | 1(≥3) |  |
| Fever | 1(1) | 1(1-2) |  | 1(1) | 2(NA) | 1(1) |  | 1(1), 1(2) |  |  |  |  |  |
| Periorbital edema | 1(1) |  |  | 1(1) |  |  |  |  |  |  |  |  |  |
| Rhinorrhea | 1(1) |  |  |  |  |  |  |  |  |  |  |  |  |
| Diarrhea | 3(1) |  |  |  |  |  |  |  |  |  |  |  |  |
| Dysphagia | 1(2) | 2(1-2) |  |  |  |  |  |  |  |  |  |  |  |
| SIADH | 1(1) |  |  |  |  |  |  |  |  |  |  |  |  |
| Urinary Tract Infection | 1(1), 1(3) |  |  |  |  |  |  |  |  | 1(2) |  |  |  |
| Vomiting | 1(2) |  |  | 1(1) |  |  |  |  |  | 1(2), 2(1) |  |  | 1(NA) |
| Constipation | 1(2) |  |  |  |  |  |  |  |  | 4(1), 1(2) |  |  |  |
| Nausea | 2(1) | 1(1-2) |  | 1(1) |  |  |  | 1(1), 1(2) |  | 2(1) |  |  | 1(NA) |
| Urinary retention | 1(2) |  |  | 1(1) |  |  |  |  |  | 1(2) |  |  |  |
| Intermittent diarrhea | 1(1) |  |  |  |  |  |  |  |  |  |  |  |  |
| Arrhythmia | 1(2) |  |  |  |  |  |  |  |  |  |  |  |  |
| Hypotension | 3(2) |  |  |  | 1(NA) |  | 2(3-4) |  |  | 1(2) |  |  |  |
| Atrial fibrillation | 1(2) |  |  |  |  |  |  |  |  |  |  |  |  |
| Sinus bradycardia | 1(1) |  |  |  |  |  |  |  |  | 1(1) |  |  |  |
| Hypertension | 1(2) | 1(1-2) |  | 1(3) |  |  |  |  |  |  |  |  | 3(NA) |
| Orthostatic hypotension | 1(2) |  |  |  |  |  |  |  |  |  |  |  |  |
| Thrombolytic event | 1(2) |  |  |  |  |  |  |  |  |  |  |  |  |
| Bradycardia | 1(1) |  |  |  |  |  |  |  |  |  |  |  |  |
| Hypoxia | 2(2) |  |  |  |  |  |  |  |  | 1(2), 1(3) |  |  |  |
| Head strike injury | 1(1) |  |  |  |  |  |  |  |  |  |  |  |  |
| Limb edema | 1(1), 1(1) |  |  |  |  |  |  |  |  | 2(1) |  |  | 1(NA) |
| Elevated alkaline phosphatase | 1(1) |  |  | 1(1) |  |  |  |  |  |  |  |  |  |
| Elevated ALT | 1(3) |  |  |  |  |  |  |  |  | 1(1) |  |  |  |
| Elevated AST | 1(3) |  |  |  |  |  |  |  |  |  |  |  |  |
| Hypoalbuminemia | 1(2) |  |  |  |  |  |  |  |  | 1(1) |  |  |  |
| Hypokalemia | 2(1) |  |  |  |  |  |  |  |  | 2(1) |  |  |  |
| Hypophosphatemia | 2(2) |  |  | 1(2) |  |  |  |  |  | 1(2), 1(1) |  |  |  |
| Hyponatremia | 1(1) |  |  |  |  |  |  |  |  | 1(1), 2(2) |  |  |  |
| Hypomagnesemia | 1(1) |  |  |  |  |  |  |  |  |  |  |  |  |
| Cerebral edema |  | 1(3-4) |  |  |  |  |  | 2(4) |  |  |  |  |  |
| Cognitive disturbance |  | 1(1-2) |  | 1(3) |  |  |  |  |  |  |  |  | 1(NA) |
| Dysphasia |  | 1(1-2) |  | 1(3) |  |  |  | 1(2) |  | 1(2) |  |  | 1(NA) |
| Facial muscle weakness |  | 1(1-2) |  |  |  |  |  | 1(3) |  | 1(1) |  |  |  |
| Muscle weakness left-sided |  | 1(3-4) |  | 1(2), 1(3), 1(2) | |  |  | 1(2), 1(3) |  |  |  |  |  |
| Seizure |  | 1(3-4) |  | 2(1) |  |  |  | 2(3) |  | 1(1) |  |  |  |
| Depression |  | 1(1-2) |  |  |  |  |  |  |  |  |  |  |  |
| Thromboembolic event |  | 1(1-2) |  |  |  |  |  |  |  |  |  |  |  |
| Breath odor-creamed corn |  | 1(1-2) |  |  |  |  |  |  |  |  |  |  |  |
| Gait disturbance |  | 1(1-2) |  | 1(3) |  |  |  |  |  |  |  |  | 2(NA) |
| Fall |  | 4(1-2), 4(1-2) |  |  |  |  |  |  |  | 1(1) |  |  |  |
| Hyperthyroidism |  | 1(1-2) |  |  |  |  |  |  |  |  |  |  |  |
| Hyperglycemia |  | 1(1-2) |  |  |  |  |  |  |  |  |  |  | 1(NA) |
| Hypernatremia |  | 1(1-2) |  |  |  |  |  |  |  |  |  |  |  |
| Pruritus |  | 1(1-2) |  | 1(1) |  |  |  | 1(2) |  |  |  |  |  |
| Mild odour |  | 1(1-2) |  |  |  |  |  |  |  |  |  |  |  |
| Pain in extremity |  | 2(1-2) |  |  |  |  |  |  |  |  |  |  |  |
| Generalized muscle weakness |  | 1(1-2) |  |  |  |  |  | 1(2) |  | 1(1), 1(3) |  |  | 1(NA) |
| Muscle weakness lower limb |  | 1(1-2) |  | 1(2) |  |  |  | 1(1) |  |  |  |  |  |
| Lung infection |  | 1(3-4) |  |  |  |  |  |  |  |  |  |  |  |
| Lactic acidosis |  | 1(1-2) |  |  |  |  |  |  |  |  |  |  |  |
| Surgery fluid shifts |  | 1(1-2) |  |  |  |  |  |  |  |  |  |  |  |
| Confusion |  |  |  | 1(3), 2(1) |  |  |  |  |  |  |  |  | 1(NA) |
| Insomnia |  |  |  | 1(2) |  |  |  |  |  | 1(1) |  |  | 1(NA) |
| Restlessness |  |  |  | 1(1) |  |  |  |  |  |  |  |  |  |
| Depressed level of consciousness |  |  |  | 1(3) |  |  |  |  |  |  |  |  |  |
| Dysarthria |  |  |  | 1(1) |  |  |  | 1(2) |  |  |  |  |  |
| Extrapyramidal disorder |  |  |  | 1(1) |  |  |  |  |  |  |  |  |  |
| Hemiparesis right sided |  |  |  | 2(3) |  |  |  |  |  |  |  |  |  |
| Ischemia cerebrovascular |  |  |  | 1(2) |  |  |  |  |  |  |  |  |  |
| Lethargy |  |  |  | 1(3) |  |  |  | 1(2) |  |  |  |  |  |
| Memory impairment |  |  |  | 1(1) |  |  |  |  |  |  |  |  |  |
| Necrosis of CNS |  |  |  | 1(2) |  |  |  |  |  |  |  |  |  |
| Somnolence |  |  |  | 1(3) |  |  |  |  |  | 1(2) |  |  |  |
| Stroke |  |  |  | 1(3) |  |  |  |  |  |  |  |  |  |
| Syncope |  |  |  | 1(3) |  |  |  |  |  |  |  |  |  |
| Injection site reaction |  |  |  | 1(1) |  |  |  |  |  |  |  |  |  |
| Anorexia |  |  |  | 1(3), 1(2) |  |  |  |  |  | 1(3) |  |  |  |
| Flushed/redness |  |  |  | 1(1) |  |  |  |  |  | 1(1) |  |  | 1(NA) |
| Hot flashes |  |  |  | 1(1) |  |  |  |  |  |  |  |  |  |
| Fecal incontinence |  |  |  | 1(1) |  |  |  |  |  |  |  |  |  |
| Abdominal distension |  |  |  | 1(2) |  |  |  |  |  |  |  |  |  |
| Dyspepsia |  |  |  | 1(1) |  |  |  |  |  |  |  |  |  |
| Sinus tachycardia |  |  |  | 3(1) |  |  |  |  |  |  |  |  |  |
| Muscle weakness right-sided |  |  |  | 1(3) |  |  |  | 3(2) |  |  |  |  |  |
| Muscle weakness upper limb |  |  |  | 1(3) |  |  |  |  |  |  |  |  |  |
| Lymphocyte count decreased |  |  |  | 1(2), 1(3) |  |  | 18(3-4) |  |  | 3(3) |  | 1(≥3) | 1(NA), 1(NA) |
| Platelet count decreased |  |  |  | 1(1) |  |  | 18(3-4) |  |  | 1(1) |  |  | 1(NA) |
| Pulmonary edema |  |  |  |  | 2(NA) |  |  |  |  |  |  |  |  |
| Epileptic events |  |  |  |  |  | 1(NA) |  |  |  |  |  |  |  |
| Olfactory auras |  |  |  |  |  | 1(NA) |  |  |  |  |  |  |  |
| Transient motor weakness |  |  |  |  |  |  | 1(3-4) |  |  |  |  |  |  |
| Non-specific neurological/suspected seizure |  |  |  |  |  |  | 10(2) | 1(2) |  |  |  |  |  |
| Febrile neutropenia |  |  |  |  |  |  | 2(3-4) |  |  |  |  |  |  |
| Asymptomatic bacteremia |  |  |  |  |  |  | 8(3-4) |  |  |  |  |  |  |
| Prolonged PTT |  |  |  |  |  |  | 1(3-4) |  |  |  |  |  |  |
| Deep vein thrombosis |  |  |  |  |  |  | 1(3-4) |  |  |  |  |  |  |
| Anemia |  |  |  |  |  |  | 9(3-4) |  |  | 1(1) |  |  | 1(NA) |
| Neutropenia |  |  |  |  |  |  | 18(3-4) |  |  |  |  |  |  |
| Transaminitis |  |  |  |  |  |  | 2(3-4) |  |  |  |  |  |  |
| Transient urinary incontinence |  |  |  |  |  |  | 1(3-4) |  |  |  |  |  |  |
| Dyspnea |  |  |  |  |  |  | 1(3-4), 1(5) |  |  |  |  |  |  |
| Capillary leak syndrome |  |  |  |  |  |  | 1(3-4) |  |  |  |  |  |  |
| Pulmonary embolism |  |  |  |  |  |  | 1(3-4) |  |  |  |  |  |  |
| Dizziness |  |  |  |  |  |  |  | 1(1) |  | 1(2) |  |  | 1(NA) |
| Dysgeusia |  |  |  |  |  |  |  | 8(1) |  |  |  |  |  |
| Facial nerve disorder |  |  |  |  |  |  |  | 1(2) |  |  |  |  |  |
| Hypoglossal nerve disorder |  |  |  |  |  |  |  | 1(2) |  |  |  |  |  |
| Intracranial hemorrhage |  |  |  |  |  |  |  | 1(3) |  |  |  |  |  |
| Flu-like symptoms |  |  |  |  |  |  |  | 2(1) |  |  |  |  |  |
| Hyperhidrosis |  |  |  |  |  |  |  | 1(1) |  |  |  |  |  |
| Palmar-plantar erythrodysesthesia syndrome |  |  |  |  |  |  |  | 1(2) |  |  |  |  |  |
| Optic nerve disorder |  |  |  |  |  |  |  | 1(1) |  |  |  |  |  |
| Left ventricular systolic dysfunction |  |  |  |  |  |  |  | 1(3) |  |  |  |  |  |
| Cough |  |  |  |  |  |  |  | 2(1), 1(2) |  |  |  |  |  |
| Sore throat |  |  |  |  |  |  |  | 3(1) |  |  |  |  |  |
| Atelectasis |  |  |  |  |  |  |  | 1(2) |  |  |  |  |  |
| Scalp pain |  |  |  |  |  |  |  | 1(1) |  |  |  |  |  |
| Shuffling gait and tongue deviation to the left |  |  |  |  |  |  |  |  |  |  |  | 1(≥3) |  |
| Intermittent double vision |  |  |  |  |  |  |  |  |  |  |  |  | 1(NA) |
| Vitreous hemorrhage |  |  |  |  |  |  |  |  |  |  |  |  | 1(NA) |
| Fungus of foot and groin |  |  |  |  |  |  |  |  |  |  |  |  | 1(NA) |
| Ringworm |  |  |  |  |  |  |  |  |  |  |  |  | 1(NA) |
| Hypocalcemia |  |  |  |  |  |  |  |  |  |  |  |  | 1(NA) |
| Delirium |  |  |  |  |  |  |  |  |  | 1(2) |  |  |  |
| Tremor |  |  |  |  |  |  |  |  |  | 1(1) |  |  |  |
| Urinary incontinence |  |  |  |  |  |  |  |  |  | 1(2), 1(1) |  |  |  |
| Skin ulceration (sacral pressure ulcer) |  |  |  |  |  |  |  |  |  | 1(3) |  |  |  |
| Hypothermia |  |  |  |  |  |  |  |  |  | 1(2) |  |  |  |
| Urinary urgency |  |  |  |  |  |  |  |  |  | 1(2) |  |  |  |
| Voice alteration |  |  |  |  |  |  |  |  |  | 1(2) |  |  |  |
| Flatulence |  |  |  |  |  |  |  |  |  | 1(1) |  |  |  |
| Hiccups |  |  |  |  |  |  |  |  |  | 1(1) |  |  |  |
| Flank pain |  |  |  |  |  |  |  |  |  | 1(1) |  |  |  |
| Decreased fibrinogen |  |  |  |  |  |  |  |  |  | 4(1) |  |  |  |
| Increased lipase |  |  |  |  |  |  |  |  |  | 1(1), 3(2) |  |  |  |
| Increased serum amylase |  |  |  |  |  |  |  |  |  | 4(1) |  |  |  |
| Increased lactic acid |  |  |  |  |  |  |  |  |  | 1(1) |  |  |  |

Supplemental 4: Side effects, summarised by severity of side effect from 1 to 5, per study. NA = severity not reported by study.

| **Study** | **1-2** | **3-4** | **5** | **NA** |
| --- | --- | --- | --- | --- |
| Bagley 2024.1 | 27 | 5 | 0 | 0 |
| Bagley 2024.2 | 48 | 7 | 0 | 1 |
| Brown 2015 | 0 | 4 | 0 | 0 |
| Brown 2022 | 33 | 18 | 0 | 0 |
| Choi 2024 | 41 | 5 | 0 | 0 |
| Goff 2019 | 1 | 15 | 1 | 0 |
| Guo 2019 | 2 | 0 | 0 | 2 |
| Landi 2023 | 0 | 0 | 0 | 22 |
| Lin 2021 | 1 | 0 | 0 | 3 |
| O’Rourke 2017 | 28 | 7 | 0 | 0 |
